# A multiplatform metabolomic approach to characterize fecal signatures of negative postnatal events in chicks: a pilot study

**DOI:** 10.1186/s40104-019-0335-8

**Published:** 2019-04-09

**Authors:** Stéphane Beauclercq, Antoine Lefèvre, Frédéric Montigny, Anne Collin, Sophie Tesseraud, Christine Leterrier, Patrick Emond, Laurence A. Guilloteau

**Affiliations:** 1 BOA, INRA, Université de Tours, 37380 Nouzilly, France; 2 Université de Tours, PST Analyse des systèmes biologiques, Tours, France; 3 PRC, INRA, CNRS, Université de Tours, IFCE, 37380 Nouzilly, France; 4 UMR 1253, iBrain, Université de Tours, Inserm, Tours, France; 5 CHRU de Tours, Service de Médecine Nucléaire In Vitro, Tours, France

**Keywords:** Biomarker, Chick, Feces, GC-MS, LC-HRMS, Negative postnatal event

## Abstract

**Background:**

Negative experiences in early life can induce long-lasting effects on the welfare, health, and performance of farm animals. A delayed placement of chicks in rearing houses has negative effects on their performance, and results in fecal-specific odors detectable by rats. Based on this observation, the volatile organic compounds (VOCs) and metabolites from the feces of 12-day-old chickens were screened for early markers of response to negative events using gas-chromatography and liquid-chromatography coupled with mass spectrometry (GC-MS, LC-HRMS).

**Results:**

The low reproducibility of solid-phase micro-extraction of the VOCs followed by GC-MS was not suitable for marker discovery, in contrast to liquid extraction of metabolites from freeze-dried feces followed by GC-MS or LC-HRMS analysis. Therefore, the fecal metabolome from 12-day-old chicks having experienced a normal or delayed placement were recorded by GC-MS and LC-HRMS in two genotypes from two experiments. From both experiments, 25 and 35 metabolites, respectively explaining 81% and 45% of the difference between delayed and control chickens, were identified by orthogonal partial least-squares discriminant analysis from LC-HRMS and GC-MS profiling.

**Conclusion:**

The sets of molecules identified will be useful to better understand the chicks’ response to negative events over time and will contribute to define stress or welfare biomarkers.

**Electronic supplementary material:**

The online version of this article (10.1186/s40104-019-0335-8) contains supplementary material, which is available to authorized users.

## Background

The perinatal period is a critical period for livestock animals. Recent studies have highlighted that the stress experienced during the perinatal period has long-term consequences on the health and the welfare of broilers [1]. After hatching, the chicks can be exposed to various factors such as temperature variations, confinement, and movement or vibrations during transportation without access to water and feed for up to 3 d. This postnatal environment can influence the behavior of adult broilers [2], their performance (i.e. body weight, feed conversion ratio, *Pectoralis major* muscle weight) [3, 4], their susceptibility to diseases that could lead to death [5], which impacts animal welfare with economic and social issues to be considered for the sustainability of poultry rearing industry. Therefore, the early detection of persisting responses to stressful events has become a major challenge for appropriate animal health and welfare management. The terminology of stressful or negative events was used to qualify the postnatal treatment lived by chicks whether it resulted in long lasting effects or not.

Easy, non-invasive methods to detect a persisting response to stressful events are currently lacking in poultry. However, new perspectives are opening up with the development of research on body odor related to physiological status or health issues, and metabolomics research more generally. For instance, the analysis of volatile organic compounds (VOC) can help, among others, in the diagnosis of colorectal cancer (blood, urine, feces, and breath) [6], irritable bowel syndrome (breath) [7], psychological stress (skin) in humans [8] as well as *Campylobacter* (feces) infection in chickens [9]. The analysis of small polar or lipophilic non-volatile molecules by nuclear magnetic resonance or mass spectrometry metabolomics is another powerful means to non-invasively detect subtle phenotypic changes, which are critical for farm management, but this method has rarely been applied in livestock studies [10]. However, some advances have recently been achieved in chickens to predict or to have a better understanding of quality or digestive physiology issues using blood, muscle, and digestive content metabolomes [11–13].

Exposures to stressful situations alter the gut microbiota composition and impact the composition of the feces as well as their odor, which in the case of rodents can be distinguished by conspecifics and cause avoidance [14, 15]. The smelling ability of rats holds promise as a tool for detecting stress or health issues and has inspired the development of electronic noses. For example, trained rats are at least as sensitive as the conventional Ziehl-Neelsen stain for detecting tuberculosis in sputum [16]. Metabolomes are currently not exploited for stress diagnosis in mammals or birds, with the exception of measuring glucocorticoid metabolites in blood or feces [17, 18], despite their usefulness in assessing the consequences of stressful events on the global metabolism.

An experimental model reproducing adverse perinatal conditions in chicks was previously developed and the potential of rats to distinguish exposed chicks based on their fecal odors was tested with success [14]. In fact, Bombail et al. highlighted a behavior change in the rats in the presence of chicken feces collected 12 d after the birds had been exposed to a negative postnatal experience, which may confirm the existence of stress-specific odors in poultry [14].

The aim of the present pilot study was thus to evaluate the potential of fecal VOC and metabolites in chicks at 12 d as early signatures of a negative postnatal experience. Due to the low reproducibility of VOC capture, the fecal metabolome acquired by GC-MS and LC-HRMS containing fewer volatile molecules was chosen for study. The chick fecal metabolomes (i.e. fecal matter and urine) were studied in two different genotypes of broilers to identify a potential typical metabolic signature that could be associated with a negative postnatal experience. This is part of a first attempt to identify potential biomarkers of value for early signatures of this experience.

## Methods

All chemicals were bought from Sigma Aldrich (Saint-Quentin Fallavier, France) unless otherwise specified.

### Birds and sample collection

Chicks randomly selected were either directly placed in the experimental rearing facility after their withdrawal from the incubator (control group or C) or subjected to a delay period before their placement (delayed group or D) to be compared for the analysis of the consequences of this negative experience as described previously [19]. The delayed group was deprived of feed and water and put in transportation boxes under irregular movement and variable room temperature: 32 °C (30 min), 21 °C (90 min), 32 °C (30 min), and then at 21 °C with alternated cycles of box movement (M) and immobility (I) for 24 h after hatching. One cycle was 45 min (M), 15 min (I), 30 min (M), 30 min (I) [19]. The D and C chicks were thereafter reared together in separated pens (1 m × 3 m) in the same room at the Pôle d’Expérimentation Animale de Tours (PEAT) (INRA Centre Val de Loire, France) in floor pens in standard temperature and light conditions (16 h light and 8 h darkness) with ad libitum access to water and feed (metabolizable energy = 12.8 MJ/kg, crude protein = 22%).

The chicks (males and females) originated from Hubbard Classic genotype (Quintin, France) in experiment 1 and from the Ross 308 (Aviagen, Angers, France) genotype in experiment 2, which are both standard fast-growing broiler lines [20]. The Hubbard Classic cohort was composed of 13 males (6 delayed and 7 controls) and 12 females (6 delayed and 6 controls), and the Ross 308 cohort included 11 males (6 delayed and 5 controls) and 12 females (6 delayed and 6 controls). The feces (including urine) of control (C) and delayed fed (D) chicks were sampled individually within sterile petri dishes in clean buckets at 12 d of age (11 d after delayed placement), 2 h after lighting was on, then packaged in closed 2 mL glass vials and frozen (− 80 °C) before VOC or fecal metabolite extraction. Chicken body weight was measured at 12 d of age.

### Fecal volatilome

The VOC extraction parameters were optimized using a pool of feces from 180 chicks from the Hubbard Classic control group.

#### Solid-phase microextraction (SPME)

Headspace SPME [21] was performed on 400 mg of fresh feces enclosed in a 2-mL glass vial. The extraction protocol was optimized by testing 4 extraction temperatures (20 °C, 40 °C, 60 °C, and 80 °C) and 3 exposure times (30 min, 1 h, 2 h) using 100 μm polydimethylsiloxane (PDMS) non-bonded fiber. Furthermore, 5 other SPME fibers (30 μm PDMS, 7 μm PDMS, 75 μm carboxen (CAR)/PDMS, 65 μm PDMS/divinylbenzene, and 85 μm polyacrylate) from Supelco (Bellefonte, PA) were tested for the maximum number of VOC recovered. The optimal extraction parameters were 1 h at 60 °C under magnetic agitation using the 85 μm polyacrylate fiber, which is designed for capturing polar semi-volatile molecules (MW 80–300). The analytical variability of VOC extraction and GC-MS analysis was assessed by calculating the variation of total feature intensities among 3 replicate samples.

#### GC-MS analyses

A Shimadzu GC-MS system (Kyoto, Japan) was used. It was composed of an AOC-20S auto-sampler, an AOC-20i auto-injector, a gas chromatograph 2010, and a QP-2010-Plus mass spectrometer. The VOC samples were desorbed from the fibers in the injection port at 250 °C and separated on a Zebron capillary ZB-5, 15 m × 0.25 mm i.d., 0.25 μm film thickness GC column (Phenomenex, Torrance, CA). The oven temperature was set at 50 °C for 1 min, ramped to 250 °C at 10 °C/min and then held for 5 min. Helium was used as the carrier gas and set at 1.2 mL/min. The ion source and interface temperature were 200 °C and 250 °C, respectively. The mass spectra of all GC peaks were generated by electron impact (EI) at 70 eV and recorded in a positive total ion monitoring mode scanning the 35–500 *m/z* range (event time = 0.2).

### Fecal metabolome

Specific preanalytical protocols were adapted from Diémé et al. including metabolome extraction followed by chemical derivatization for GC-MS analysis [22].

#### GC-MS

Ten mg of freeze-dried chick feces were put into 2 mL acidified salt water (120 mmol/L HCl, 0.9% NaCl), followed by vortexing and centrifugation (3,000×*g*, 20 °C). The metabolites were further extracted from the mix by 2 mL of ethyl acetate. The organic phase was recovered and evaporated in a SpeedVac (Thermo Fisher Scientific, Waltham, MA) at room temperature. Each sample was derivatized by the addition of 70 μL of a mixture of N,O-bistrifluoroacetamide (BSTFA) and trimethylchlorosilane (TMCS; BSTFA/TMCS: 99/1), and 30 μL of acetonitrile for 40 min at 80 °C in a sand bath. A volume of 1 μL of the derivatized mixture was injected in the GC-MS system previously described with the oven temperature set at 80 °C for 3 min, ramped to 250 °C at 5 °C/min and then held for 25 min. The carrier gas (He) flow was set at 1.0 mL/min. The instrumental stability and extraction reproducibility were evaluated by multiple injections (*n* = 6) of a quality control (QC) sample obtained from the extraction of 10 mg from a pool of all samples analyzed.

#### LC-HRMS

Three mg of freeze-dried chick feces were put into 500 μL of a methanol/water mix (8:2) and vortexed for 2 min. The mixes were centrifuged (15,000× *g*, 15 min, 4 °C) to sediment solid particles, then 450 μL of the supernatant was recovered and put in glass tubes for further solvent evaporation in a SpeedVac at room temperature. The residues were then reconstituted with 200 μL methanol/water (1:1) followed by centrifugation (20,000× *g*, 10 min, 4 °C) before Ultra-High-Performance Liquid Chromatography (UHPLC) separation and mass spectrometry analysis. LC-HRMS analysis was performed on a UHPLC Ultimate 3000 system (Dionex, Sunnyvale, CA), coupled to a Q-Exactive mass spectrometer (Thermo Fisher Scientific) and operated in positive (ESI+) and negative (ESI–) ionization modes. Chromatography was carried out with a 1.7-μm XB – C18 (150 mm × 2.10 mm, 100 Å) UHPLC column (Kinetex, Phenomenex, Torrance, CA) heated at 40 °C. The solvent system comprised mobile phase A [water + 0.1% (*v*/*v*) formic acid], and mobile phase B [methanol + 0.1% (*v*/*v*) formic acid]; the gradient operated at a flow rate of 0.4 mL/min over a run time of 24 min. The multistep gradient was programmed as follows: 0–1.5 min, 32–45% A; 1.5–5 min, 45–52% A; 5–8 min, 52–58% A; 8–11 min, 58–66% A; 11–14 min, 66–70% A; 14–18 min, 70–75% A; 18–21 min, 75–97% A; 21–24 min, 97% A. The autosampler (Ultimate WPS-3000 UHPLC system, Dionex, Sunnyvale, CA) temperature was set at 4 °C, and the injection volume for each sample was 5 μL. Heated ESI source parameters were a spray voltage of 3.5 kV, capillary temperature of 350 °C, heater temperature of 250 °C, sheath gas flow of 35 arbitrary units (AU), auxiliary gas flow of 10 AU, spare gas flow of 1 AU, and tube lens voltage of 60 V for C18. During the full-scan acquisition, which ranged from 58 to 870 *m/z*, the instrument operated at 70,000 resolution (*m/z* = 400), with an automatic gain control target of 1 × 10^6^ charges and a maximum injection time of 250 ms. The instrumental stability was evaluated by multiple injections (*n* = 6) of a QC sample obtained from a pool of 10 μL of all samples analyzed. This QC sample was injected once at the beginning of the analysis, every 10 sample injections, and at the end of the run.

### Data processing and spectral assignment

#### GC-MS

Each chromatogram obtained was processed for smoothing, library matching, and area calculation using the GC-MS Solution Postrun Analysis software (Shimadzu, Japan) as described in Emond et al. [23]. To minimize process errors, each integrated peak was manually checked for each sample and features with greater than 30% variability in QC samples were rejected [24]. Compounds were identified from their electron impact mass spectra by comparison to our in-house library (250 molecules) combined with the NIST spectral mass library (NIST 05), HMDB [25], and ChemSpider. This annotation reached level 2 on the scale of confidence in metabolite identification, as defined by the Chemical Analysis Working Group of the Metabolomics Standards Initiative [26]. The feature areas were normalized by dividing each one by the total area of the chromatogram and by the weight of each dried sample.

#### LC-HRMS

A library of standard compounds (Mass Spectroscopy Metabolite Library of standards MS ML®, IROA technologies) was analyzed with the same gradient of mobile phases and in the same conditions as those used to analyze feces samples. The annotation of selected features was validated from retention time, and high-resolution mass molecular. Targeted molecules (367 molecules detected in ESI+, 255 in ESI-) were selected and integrated into Xcalibur 2.2 (Thermo Fisher Scientific, San Jose, CA) as described by Bitar et al. [27]. This annotation reached level 1 on the scale of confidence in metabolite identification [26]. Each peak area was normalized to the total peaks area of each chromatogram and by the weight of each dried sample. Peaks with greater than 30% variability in QC samples were rejected as unsuitable for further investigation.

### Chemometry and statistical analysis

The effects of the delayed placement on body weight were analysed by ANOVA after having checked the normality of data distribution (XLSTAT software, Addinsoft, Paris, France).

Principal Component Analyses (PCA) were performed on the variance unit scaled data sets from the GC-MS and LC-HRMS fecal metabolome analyses as an exploratory unsupervised analysis. Individuals outside of the 95% confidence interval of the Hotelling’s T-square in the GC-MS and LC-HRMS experiments were considered to be outliers and excluded from the subsequent analyses [28].

An orthogonal projection to latent structures discriminant analysis (OPLS-DA) was performed using the SIMCA 13 Software (version 13.0, Umetrics, Umeå, Sweden) on the two data sets (i.e., GC-MS, LC-HRMS). All data were scaled to unit variance to maximize the separation between the delayed and control groups. OPLS-DA is a method of supervised classification that predicts the categorical factor *Y* (C or D group) by explanatory quantitative variables *X* (metabolites). OPLS-DA modeling was chosen instead of partial least squares discriminant analysis (PLS-DA) for its inclusion of orthogonal components containing variations that do not contribute to the discrimination between the control and delayed groups. Variation in the spectral data *X* is divided into one predictive component containing variations correlated with the class identifier *Y* and single or multiple orthogonal components containing variations orthogonal to the predictive component that do not contribute to discrimination between the defined groups [29]. The minimum number of features needed for optimal classification of the OPLS-DA models was determined by iteratively excluding the variables with low regression coefficients and wide confidence intervals derived from jackknifing combined with low variable importance in the projection (VIP) until maximum improvement of the quality of the models. The metabolite with the lowest *P*-value (Welch’s means equality *t*-test) was conserved in the model if the same metabolite was detected in the positive and negative ionization mode for LC-HRMS. The model quality was evaluated after 7-fold cross validation by cumulative R^2^Y (goodness of fit), cumulative Q^2^ (goodness of prediction), and CV-ANOVA (cross validation-analysis of variance). CV-ANOVA is a diagnostic tool for assessing the reliability of OPLS-DA models; the returned P-value is indicative of the statistical significance of the fitted model [30]. The contribution of each predictor in the model was evaluated through the variable score contribution (i.e. the differences, in scaled units, for all the terms in the model, between the outlying observation and the normal observation, multiplied by the absolute value of the normalized weight) and the importance in the model (VIP). Subsequently, the OPLS-DA models, as described above, were fitted on the GC-MS and LC-HRMS data sets, considering the genotypes (i.e. Hubbard Classic, Ross 308) independently.

The biochemical information about the metabolites retained in the OPLS-DA models that was used to develop the discussion was partly extracted from the HMDB database [25] and KEGG [31].

## Results

### Effect of delayed placement on chicken growth

**Fig. 1:**
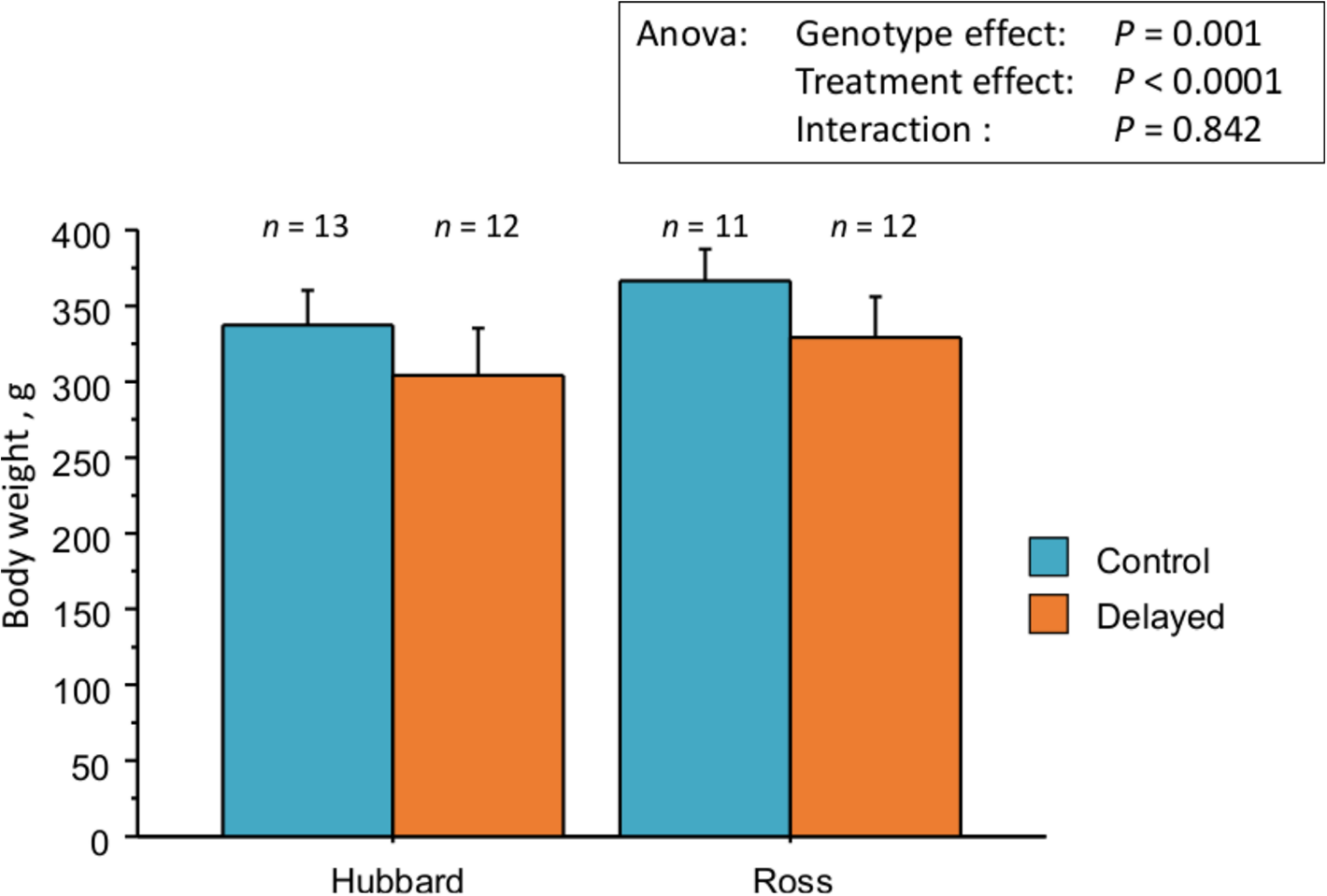
Effect of delayed placement on chicken body weight. The histograms indicated the mean and standard deviation of the delayed (D) and the control (C) groups at 12 d of age. The statistical analysis of the different effects is indicated on the figure

The body weight of chicks at 12 d of age was significantly impacted by the genotype (*P* = 0.001) and by the delayed placement in rearing facilities for both genotypes (*P*< 0.0001) (Fig. 1). There were significant reductions by 9.7% and 9.8% of the average body weight between the control (C) and the delayed (D) for Hubbard Classic and Ross 308 genotypes, respectively. Those differences were conserved until slaughter (34 d) as previously reported [19]. In these experimental conditions, the delayed placement did not affect the welfare and the prevalence of health disorders [19]. The feces from all the animals were sampled individually for fecal volatilome and metabolome analyses at 12 d of age for the control and delayed chick groups.

### Fecal volatilome

The extraction time and temperature were tested on a pool of feces from the Hubbard Classic genotype chicks using 100 μm polydimethylsiloxane SPME fiber designed to capture volatile molecules (red, MW 60–275). It appeared from those tests that the capture of VOCs in the head space at 60 °C for 1 h resulted in a GC-MS chromatogram containing 49 peaks. Using the same extraction conditions, the number of peaks was increased to 56 and 93 using 65 μm polydimethylsiloxane/divinylbenzene fiber (blue) and 85 μm polyacrylate fiber (white), respectively. Those fibers are designed to capture volatiles, amines, and nitro-aromatic compounds (MW 50–300), and polar semi-volatile molecules (MW 80–300), respectively. It appeared from those developments that the best experimental conditions for capturing VOCs were 1 h at 60 °C using the 85 μm polyacrylate SPME fiber. The chromatogram acquired using the optimized capture method on 3 replicates of feces from Hubbard Classic control group chicks contained on average 100 features corresponding to around 30 identified molecules (Additional file 1: Table S1). However, the capture was not reproducible because the feature total area variance was 46% for the 3 replicates. The head space SPME on chick feces was not suitable for quantitative analyses because of lack of reproducibility.

### Fecal metabolome

PCA analyses of the LC-HRMS and GC-MS metabolomics data showed that the QC samples were clustered, which validates the stability of the analysis quality during the analysis campaign (data not shown). The PCA plots also revealed that one sample could be considered to be an outlier (Hubbard Classic, female, delayed) and thus was removed from the subsequent analysis. Furthermore, those PCA highlighted that the genotype of the chicks (i.e. Hubbard Classic and Ross 308) has a higher impact on the fecal metabolome than the sex or the postnatal experience effect (Additional file 1: Figure S1), although both genotypes are selected for rapid growth and lean tissue deposition [20].

A total of 91 and 139 molecules out of the 255 and 367 metabolites present in our in-house data base were detected by LC-HRMS in ESI– and ESI+, respectively, in the Hubbard Classic and Ross 308 chicks. GC-MS, which is more suitable for quantifying volatile non-polar metabolites, allowed the detection of 114 metabolites in both genotypes.

### Fecal markers of a previous negative experience

The LC-HRMS and GC-MS data were fitted to 2 OPLS-DA models, a multivariate supervised classification method aiming to identify 2 sets of metabolites discriminating the C and D chick groups and shared by both genotypes.

**Fig. 2:**
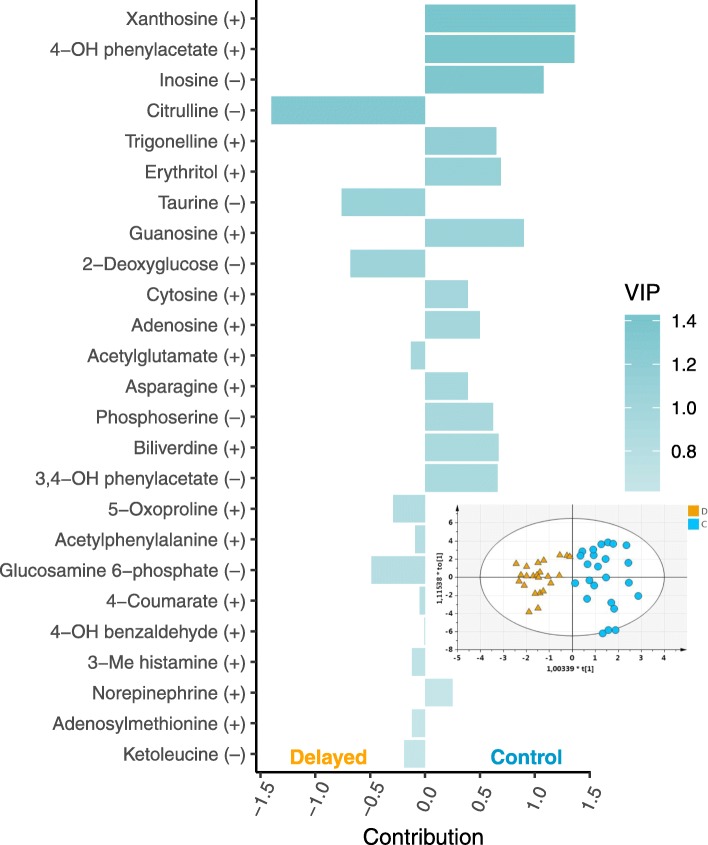
Discriminant LC-HRMS fecal metabolites (ESI+ and ESI-) and score plot based on OPLS-DA model. In the score plot, the individuals from the delayed (D) and control (C) groups are represented by orange triangles and light blue circles, respectively. The bar plot represents the contributions of the variables (a negative contribution score indicates a contribution of the variable to the delayed group while a positive score is indicative of a contribution to the control group) and their importance in projection (VIP) in the OPLS-DA model. The “+” or “–” signs after the metabolite names correspond to the LC-HRMS ionization mode. The descriptive and predictive performance characteristics of the models are R^2^Y_cum_ = 0.81 and Q^2^_cum_ = 0.73

**Fig. 3:**
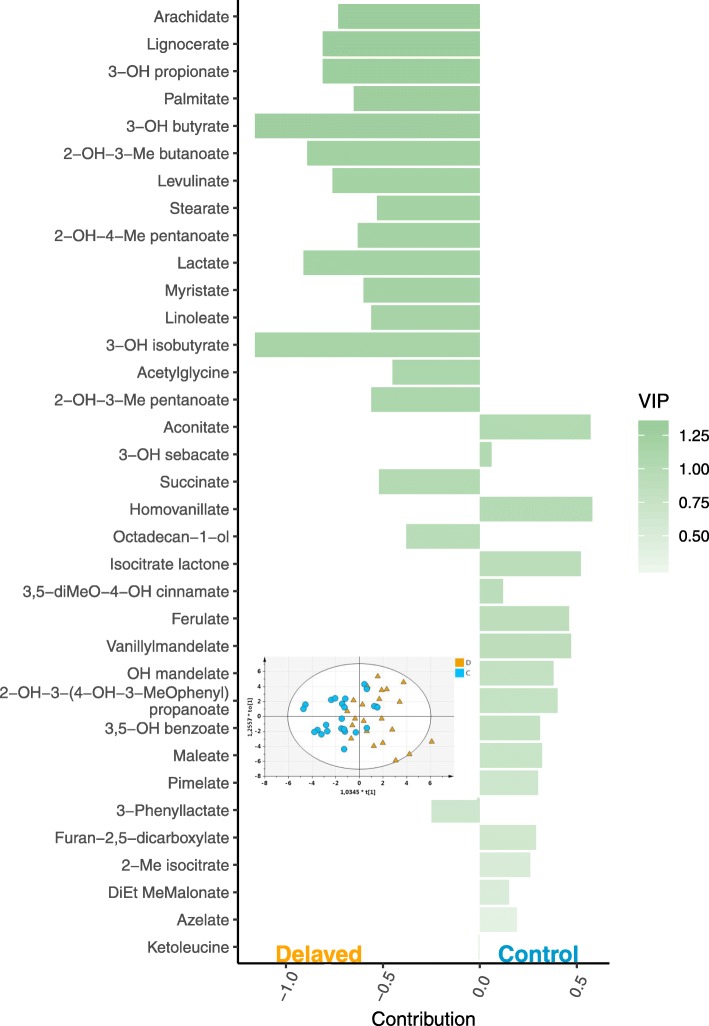
Discriminant GC-MS fecal metabolites and score plot based on OPLS-DA model. In the score plot, the individuals from the delayed (D) and control (C) groups are represented by orange triangles and light blue circles, respectively. The bar plot represents the contributions of the variables (a negative contribution score indicates a contribution of the variable to the delayed group while a positive score is indicative of a contribution to the control group) and their importance in projection (VIP) in the OPLS-DA model. The descriptive and predictive performance characteristics of the models are R^2^Y_cum_ = 0.45 and Q^2^_cum_ = 0.2

The consensus model for both genotypes based on the LC-HRMS data (ESI– and ESI+) was composed of 1 predictive and 2 orthogonal components and included 25 metabolites. This model explained 81% of the differences between the 2 groups (R^2^Y_cum_) and clearly separated the delayed and control chicks (Fig. 2). The predictive ability of this model (Q^2^_cum_) and its CV-ANOVA were 0.73 and 6.78 × 10^− 10^, respectively. The consensus model based on GC-MS data (1 predictive + 1 orthogonal component) containing 35 metabolites was less explicative (R^2^Y_cum_ = 45%) and predictive (Q^2^_cum_ = 0.2, CV-ANOVA = 0.053) than the LC-HRMS model (Fig. 3). The importance of the metabolites in both models (VIP) and their contributions are presented in Figs. 2 and 3. Only one metabolite (ketoleucine) was present in both models.

The OPLS-DA model adjusted to the metabolomics data (LC-HRMS or GC-MS) based on separately-analyzed Hubbard Classic or Ross 308 chick feces had equivalent or better explicative and predictive abilities for the groups and included fewer metabolites (i.e. 21–17) than the consensus models (Additional file 1: Figures S2 and S3). Only one metabolite (succinate) was common to the consensus, Hubbard Classic, and Ross 308 OPLS-DA models in GC-MS, which may result from different ways to deal with negative experience between the 2 genotypes.

## Discussion

### Fecal volatilome

The setup of SPME extraction conditions is tricky and species-dependent. For example, as reported by Reade et al., the optimal extraction methods are different between human and murine feces samples [32]. To our knowledge only one study of the chicken fecal volatilome has been previously done using frozen feces (60 °C, 1 h capture with carboxen/polydimethylsiloxane SPME fiber), but it did not include reproducibility information [9]. Moreover, the composition of the feces may be different between chickens and chicks. The first step was thus to define the optimal conditions to analyze the fecal volatilome. It appeared from our developments that the best experimental conditions for capturing VOCs were 1 h at 60 °C, as proposed in Garner et al., using the 85 μm polyacrylate SPME fiber [9]. The head space SPME on chick feces was not suitable for quantitative analyses because of lack of reproducibility. Liquid extraction on freeze-dried feces followed by LC-HRMS or GC-MS was preferred because of higher reproducibility. To illustrate this point, the variance of the 250 LC-HRMS features under analysis varied 14% between the 6 replicates from the pool of feces previously described.

### Fecal markers of a previous negative experience

The OPLS-DA models were a first attempt to characterize the fecal signature of postnatal negative events in chicks. Even if the relative reduction in body weight was similar between the Hubbard Classic and Ross 308 chicks, it appeared that the metabolic profiles related to negative postnatal events were different between the two genotypes (Additional file 1: Figures S2 and S3). The only metabolites (succinate) that was present in consensus and separate models cannot explain the rat behavior changes observed in a previous experiment as it is a non-volatile compound. From the Hubbard Classic (the chicks used in the Bombail et al. rat trial [14]) feces, only 2 volatile compounds were included in the OPLS-DA: cadaverine (LC-HRMS; vapor pressure = 1.0 ± 0.3 mmHg at 25 °C) and guaiacol (GC-MS; vapor pressure = 0.2 ± 0.4 mmHg at 25 °C). Cadaverine is a foul-smelling diamine formed by the decarboxylation of lysine by gut microbiota, while guaiacol is a phenolic product of lignin (grain cell walls [33] or ingested wood shavings from the litter) degradation.

### Biological integration of the consensus models

The consensus OPLS-DA model that was predictive of the postnatal conditions based on the fecal metabolome analyzed by LC-HRMS contained mainly amino acids or their by-products which may come from the diet or the gut microbiota metabolism: citrulline (arginine metabolism), phosphoserine, asparagine, acetylphenylalanine, 5-oxoproline (cyclic sub-product of glutamate), adenosylmethionine (methionine metabolism), and acetylglutamate. Nucleosides and the products of their catabolism were also present in the model (xanthosine, inosine, guanosine, adenosine, cytosine) as well as flavonoid/phenol and other compounds from the diet: 4-hydroxyphenylacetate [34], 4-coumarate (phenylpropanoids related to lignin biosynthesis and flavonoid production), erythritol (polyol), trigonelline (alkaloid produced by the metabolism of niacin present in soybeans [35]; soy is a major component of poultry feeds for protein supply), and 3,4-dihydroxyphenylacetate (phenolic acid formed from fermentation of soy flavonoid). The OPLS-DA model based on GC-MS was less explicative and predictive than the LC-HRMS one. It contained mainly organic acids (3-hydroxypropionate, 3-hydroxybutyrate, 3-hydroxyisobutyrate, lactate, succinate) and fatty acids (palmitate, linoleate, myristate, stearate, arachidate, lignocerate). The extraction using methanol/water for LC-HRMS and ethyl acetate for GC-MS may explain the difference between the set of metabolites retained in the two OPLS-DA models.

The identification of the metabolic pathways impacted by the negative postnatal experience using fecal metabolomics is rather complex because feces represent the final products of complex interactions involving the gut physiology, the gut immunity, the gut microbiota, the diet, genetics, and the environment [36]. Added to this, urine (in the form of uric acid) and feces are mixed in chickens. For example, the lactate (one of the strongest contributors to the delayed group in GC-MS) could be related to endogenous (cellular energy metabolism) or bacterial metabolism. The OPLS-DA models consensus to both genotypes and fitted to LC-HRMS and GC-MS included metabolites that may be related to an adaptive response to negative events as well as to differences in energy metabolism and microbiota composition between the delayed (D) and control (C) chick groups. Those 3 hypotheses will be discussed in the following paragraphs. The metabolites cited and their spectrum acquisition method as well as their contribution to the OPLS-DA models (i.e. negative values are indicative of a contribution of the metabolite to the D group and positive values to the C group) will be indicated.

One adrenergic hormone and neurotransmitter from the catecholamine family, norepinephrine (LC, 0.25), contributed less to the fecal metabolome of the delayed group. Norepinephrine is a neurotransmitter produced by the sympathetic nervous system, adrenal glands, and also by some gut bacteria [37, 38]. It is the primary neurotransmitter found in the gut of most animals and known to be secreted in response to stress, but also to regulate the behavior response to stressful stimuli in chicken [39]. Vanillomandelate (GC, 0.47), a product of norepinephrine catabolism, contributed less to the delayed group as well as 3,4-dihydroxyphenylacetate (LC, 0.66) and homovanilate (GC, 0.58), the intermediate and the final product of dopamine catabolism, respectively. Dopamine, another catecholamine synthetized in the brain and kidneys or released by gut bacteria [37, 38], plays a role in reward processing, but its release is also increased by stress [40, 41]. The lower level of catecholamine and its metabolites in the feces of the delayed chicks at 12 d of age suggested that the delayed birds have a lower basal release of catecholamine in standard rearing conditions compared to the control birds, which may be related to an adaptive response over time to a negative postnatal experience. To evaluate this hypothesis, it would be valuable to measure the expression of stress response-related genes and proteins in adrenal glands and the corticosteronemia at the baseline and in response to an acute stress after ACTH treatment [42]. This could be an adaptive process used by the delayed birds to attenuate their stress response as this is reported for the domesticated birds compared to their wild counterparts [43]. Furthermore, the contribution of 3 metabolites known for their antioxidant effects [44, 45], i.e. citrulline (LC, − 1.40), taurine (LC, − 0.76), and adenosylmethionine (LC, − 0.12) was higher in the delayed groups, also suggesting an adaptive response of the D chickens to negative postnatal events. The redox balance and the antioxidant status would be interesting to investigate in both delayed and control chickens.

During the first 24 h post-hatching, the energy metabolism of the 2 groups of chicks (D and C) was different and those differences may be conserved during their growth. The blood glucose and triglyceride levels were lower in the D group chicks at day one (unpublished data). The yolk sac is the main source of energy (via fatty acid oxidation) during embryonic development until exogenous feed is given in the brooder house [46]. Therefore, the delayed chicks consumed the rest of the nutrients contained in the internalized yolk sac (mostly fatty acids and proteins) for 24 h post-hatching while the control group had direct access to exogenous feed rich in carbohydrates (unpublished data). The consensus OPLS-DA models based on the fecal metabolome at 12 d suggested the persistence of a difference in energy metabolism between the 2 groups, at least concerning metabolism in the gut. The major indicator of this difference was the contribution of 3-hydroxybutyrate (GC, − 1.16) to the D group of chicks, which indicated a metabolic activity related to ketogenic amino acid degradation, and lipid β-oxidation in this group. 3-hydroxybutyrate is an important metabolic substrate for energy production in starvation response [47]. Ketogenic amino acid catabolism can also be illustrated by the contribution of ketoleucine (LC, − 0.19; GC, − 0.01) to the D chick excreta discrimination in the OPLS-DA model. This suggested a remaining effect of post-hatching starvation or an adaptive effect to lipid-rich substrates 12 d after the treatment. On the other hand, the digestive utilization of some lipids (medium to long carbon chain) by the D group was lower as evidenced by the negative contribution of palmitate (GC, − 0.65), linoleate (GC, − 0.56), myristate (GC, − 0.60), stearate (GC, − 0.53) and lignocerate (GC, − 0.81) to the OPLS-DA models. This may be also related to long-term effects of early food deprivation on hepatic lipid metabolism as reported in early feed restriction program [48]. In contrast, some amino acids contributed less to the fecal metabolome of the D group of chicks, namely asparagine (LC, 0.39), and phosphoserine (LC, 0.62), suggesting they may have been metabolized and/or used for energy purposes. Interestingly, 3-hydroxyisobutyrate (GC, − 1.16), a product of valine catabolism and a good gluconeogenic substrate [49], contributed to the fecal metabolome of the D group of chicks, suggesting enhanced amino acid catabolism that led to glucose production. Therefore, it seems that the group of D chicks exhibits a metabolic adaptation for energy production and utilization compared to the control group and that this adaptation may last more than 10 d after the 24 h post-hatch fasting. Beside this, biliverdin (LC, 0.67) contributed less to the fecal metabolome, including urine, of the D group. This compound results from the degradation of heme from hemoglobin in mammals and birds [50, 51]. This difference could be related to persisting changes in hepatic or renal function [52], in response to an early negative experience.

The contribution of guanosine (LC, 0.90; purine nucleoside), xanthosine (LC, 1.37), inosine (LC, 1.08), and adenosine (LC, 0.50) was higher in feces from the control chicks compared to the D chicks. Moreover, the xanthosine and the inosine were both in the VIP top 3 of the LC-HRMS OPLS-DA models, which suggests the importance of the pathways related to purine metabolism in the group of control chicks. The cytosine (LC, 0.39) also contributed to the fecal metabolome of the control group. However, it is impossible to determine if those molecules came from endogenous or gut microbiota metabolism. Indeed, some metabolites produced by gut microbiota explained the differences between delayed and control birds: 2-hydroxy-3-methylpentanoate (GC, − 0.56), and 2-hydroxy-4-methylpentanoate (GC, − 0.63), which are products of isoleucine and leucine catabolism, respectively, by *Clostridium difficile* for example [53, 54], and lactate (GC,–0.91; product of gut microbiota such as Lactobacillus acidophilus in relation with intestinal health [55]). These elements may suggest that the negative postnatal experience (i.e. fasting and stressful conditions) had an impact on the composition of the gut microbiota and the associated metabolome, the next step to investigate.

## Conclusion

The fecal volatilome was not informative due to the lack of reproducibility of the extraction by SPME and injection into the GC-MS in this pilot study, which may be improved by the automatization of the processes. In contrast, liquid extraction of freeze-dried feces followed by LC-HRMS or GC-MS was reproducible, and the OPLS-DA model fitted on those data highlights persisting differences in adaptive response, energy metabolism, and microbiota composition for the delayed chicks in response to the negative postnatal experience. Further integrative analyses are needed to characterize these differences in target tissues as brain, adrenal glands, liver for adaptive response and energy metabolism, and gut for microbiota composition. These conclusions bear out the interest of a multiplatform metabolomic approach to characterize the fecal signatures, and possibly of other biological tissues, of response to postnatal negative events.

## Additional file

Additional file 1: **Table S1.** Fecal VOCs annotated after headspace solid-phase microextraction (capture 1 h at 60 °C, 85 μm polyacrylate fiber) coupled with GC-MS. This volatilome was acquired on a pool of Hubbard control group chick feces. **Figure S1.** Score plots following principal component analysis for LC-HRMS (a, b, c) and GC-MS (d, e, f) fecal metabolomics. The first components (t [1]) explained 32% and 37% of the fecal metabolome variability while the second one (t [2]) explained 10 and 15% of variability in LC-HRMS and GC-MS, respectively. In the “a” and “d” score plots, Hubbard (H) individuals are represented in turquoise and Ross (R) individuals in purple. The sex of the chicks: male (M, blue) and female (F, red) are represented in the “b” and “e” score plots. The individuals from the delayed group (D, orange triangle) and control group (C, light blue circle) are represented in the “c” and “f” plots. **Figure S2.** OPLS-DA models adjusted to Hubbard (a) and Ross (b) chick fecal metabolomes at 12 d of age analyzed by **LC-HRMS**. The individuals from the delayed group (D, orange triangle) and control group (C, light blue circle) are represented in the score plots at the top of the figure. The fecal metabolites included in the Hubbard and Ross OPLS-DA models are tabulated with their variable importance in projection (VIP) and their contribution in the models. A negative contribution score indicates a contribution of the variable to the delayed group while a positive score is indicative of a contribution to the control group. The performance characteristics of each OPLS-DA model are under the metabolites tables (p = predictive component, o = orthogonal component). **Figure S3.** OPLS-DA models adjusted on Hubbard (a) and Ross (b) chick fecal metabolomes at 12 d of age analyzed by **GC-MS**. The individuals from the delayed group (D, orange triangle) and control group (C, light blue circle) are represented in the score plots at the top of the figure. The fecal metabolites included in the Hubbard and Ross OPLS-DA models are tabulated with their variable importance in projection (VIP) and their contribution in the models. A negative contribution score indicates a contribution of the variable to the delayed group while a positive score is indicative of a contribution to the control group. The performance characteristics of each OPLS-DA model are under the metabolites tables (p = predictive component, o = orthogonal component). (PDF 1250 kb)

## Abbreviations

C: Control chicks group
CAR: Carboxen
D: Delayed chicks group
EI: Electron impact
ESI: Electrospray ionization
Et: Ethyl
GC-MS: Gaseous chromatography coupled with mass spectrometry
LC-HRMS: Liquid chromatography coupled with high resolution mass spectrometry
Me: Methyl
MeO: Methyloxy
OH: Hydroxy
OPLS-DA: Orthogonal projection to latent structures discriminant analysis
PCA: Principal component analysis
PDMS: Polydimethylsiloxane
PLS-DA: Partial least squares discriminant analysis
Q^2^_cum_: Cumulative fraction of the variation of the categorical factor Y predicted by the model
QC: Quality control
R^2^Y_cum_: Cumulative fraction of the variation of the categorical factor Y explained by the model
SPME: Solid-phase microextraction
UHPLC: Ultra-high performance liquid chromatography
VIP: Variable importance in the projection
VOC: Volatile organic compound
