# Supplementary material for: A multiplatform metabolomic approach to characterize fecal signatures of negative postnatal events in chicks: a pilot study
Source: J Anim Sci Biotechnol. 2019 Apr 9;10:21. doi: 10.1186/s40104-019-0335-8 (PMC6454711; doi:10.1186/s40104-019-0335-8)
Supplement: Supplementary file 1 — Table S1. Fecal VOCs annotated after headspace solid-phase microextraction (capture 1 h at 60 °C, 85 μm polyacrylate fiber) coupled with GC-MS. This volatilome was acquired on a pool of Hubbard control group chick feces. Figure S1. Score plots following principal component analysis for LC-HRMS (a, b, c) and GC-MS (d, e, f) fecal metabolomics. The first components (t [1]) explained 32% and 37% of the fecal metabolome variability while the second one (t [2]) explained 10 and 15% of variability in LC-HRMS and GC-MS, respectively. In the “a” and “d” score plots, Hubbard (H) individuals are represented in turquoise and Ross (R) individuals in purple. The sex of the chicks: male (M, blue) and female (F, red) are represented in the “b” and “e” score plots. The individuals from the delayed group (D, orange triangle) and control group (C, light blue circle) are represented in the “c” and “f” plots. Figure S2. OPLS-DA models adjusted to Hubbard (a) and Ross (b) chick fecal metabolomes at 12 d of age analyzed by LC-HRMS. The individuals from the delayed group (D, orange triangle) and control group (C, light blue circle) are represented in the score plots at the top of the figure. The fecal metabolites included in the Hubbard and Ross OPLS-DA models are tabulated with their variable importance in projection (VIP) and their contribution in the models. A negative contribution score indicates a contribution of the variable to the delayed group while a positive score is indicative of a contribution to the control group. The performance characteristics of each OPLS-DA model are under the metabolites tables (p = predictive component, o = orthogonal component). Figure S3. OPLS-DA models adjusted on Hubbard (a) and Ross (b) chick fecal metabolomes at 12 d of age analyzed by GC-MS. The individuals from the delayed group (D, orange triangle) and control group (C, light blue circle) are represented in the score plots at the top of the figure. The fecal metabolites included in the Hubbard [file 40104_2019_335_MOESM1_ESM.pdf]

A multiplatform metabolomic approach to characterize fecal signatures of stressful postnatal events in chicks: a pilot study

Stéphane Beauclercq[†], *Antoine Lefèvre*[‡], *Frédéric Montigny*[‡], *Anne Collin*[†], *Sophie Tesseraud*[†], *Christine Leterrier*[§], *Patrick Emond*[‡,‖,#], *Laurence A. Guilloteau*[*,†]

[†]BOA, INRA, Université de Tours, 37380 Nouzilly, France

[‡]Université de Tours, PST Analyse des systèmes biologiques, Tours, France

[§]PRC, INRA, CNRS, Université de Tours, IFCE, 37380 Nouzilly, France

[‖]UMR 1253, iBrain, Université de Tours, Inserm, Tours, France

[#]CHRU de Tours, Service de Médecine Nucléaire In Vitro, Tours, France

Table S1. Fecal VOCs annotated after headspace solid-phase microextraction (capture 1 hour at 60 °C, 85µm polyacrylate fiber) coupled with GC-MS. This volatilome was acquired on a pool of Hubbard control group chick feces.

RT[a] (min)	Metabolites
1.13	CO2
6.859	2,4-Diisocyanatotoluene
6.937	2,4-Diisocyanatotoluene
7.017	2,4-Diisocyanatotoluene
7.135	2,4-Diisocyanatotoluene
7.273	2,4-Diisocyanatotoluene
8.234	n-1-Hexadecanol
8.847	4-Pyrrolidinopyridine
9.267	2,4,6-Trimethylbenzaldehyde
9.707	Calarene epoxide
10.071	(1,5,5-Trimethyl-2-methylenebicyclo[4.1.0]hept-7-yl)methanol
10.417	1-Tetradecanol
10.567	n-Eicosane
11.103	Diallyl maleate
11.979	1-Hexadecene
12.048	Pentanoic acid, 2,2,4-trimethyl-3-carboxyisopropyl, isobutyl ester
12.279	Hexadecanal
12.506	Decyl decanoate
12.718	1,3-Diphenylpropane
13.525	2-Ethylhexyl 4-hydroxybenzoate
13.79	5-Phenyldodecane
13.92	4-Phenyldodecane
14.178	3-Phenyldodecane
14.477	Ethylenic hydrocarbons
14.557	Saturated hydrocarbons
14.867	13-Phenyl-entacosane
14.987	2,4-Diphenyl-4-methyl-2(E)-pentene
15.077	4-Phenyleicosane
15.339	Diisobutyl phthalate
15.481	4-Octadecylmorpholine
15.634	n-Nonadecane
15.923	n-Hexadecanoic acid methyl ester
16.325	n-Butyl phthalate
16.659	n-Eicosane
17.042	1,3-Diphenyl-1,3-propanedione
17.121	Triterpene or polyaromatic hydrocarbons
17.228	By-product of polyaromatic hydrocarbons
17.557	4-Octadecylmorpholine
18.161	Fatty acid methyl ester

[a]Retention time

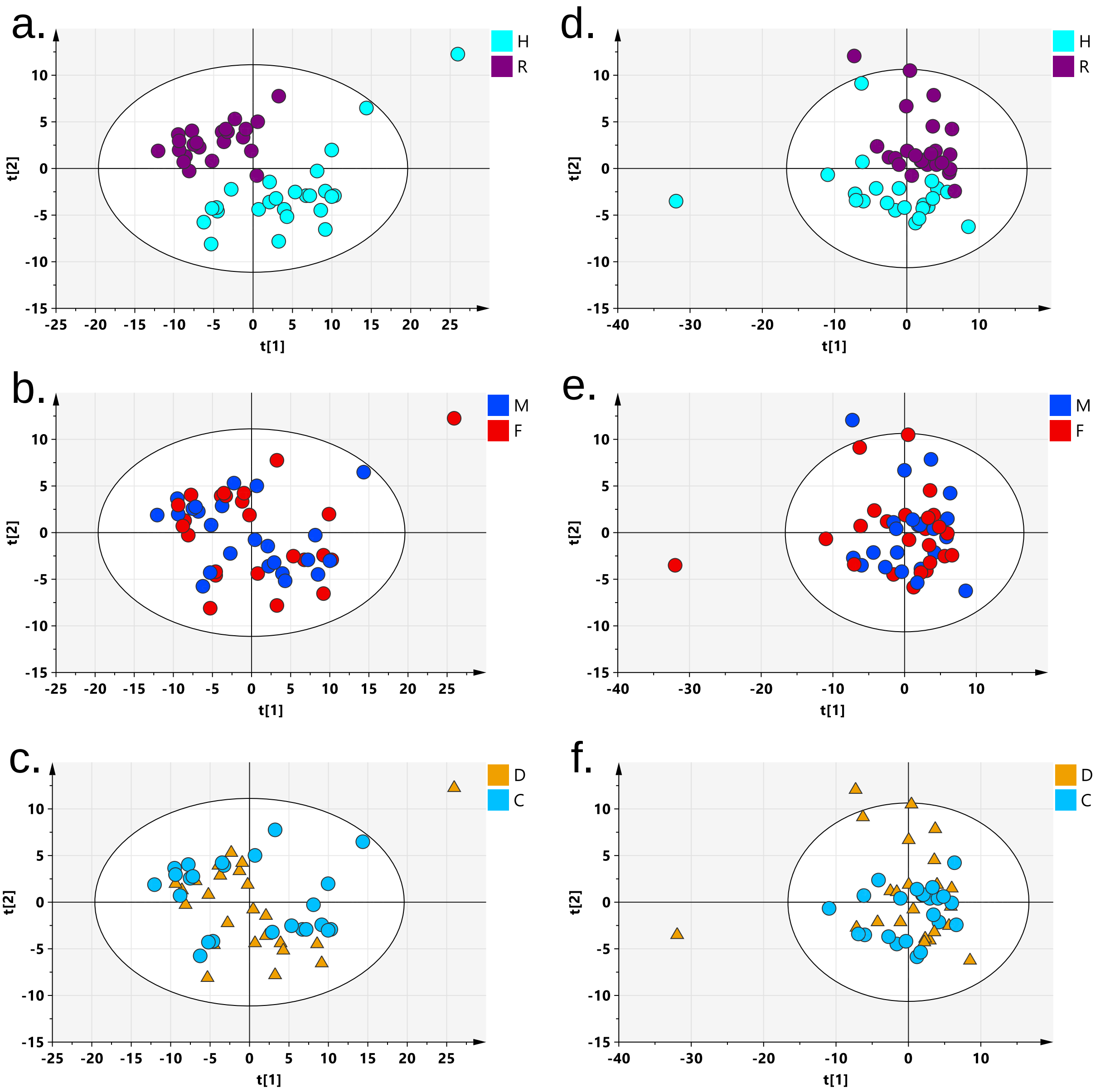

Figure S1. Score plots following principal component analysis for LC-HRMS (a, b, c) and GC-MS (d, e, f) fecal metabolomics. The first components (t[1]) explained 32% and 37% of the fecal metabolome variability while the second one (t[2]) explained 10% and 15% of variability in LC-HRMS and GC-MS, respectively. In the “a” and “d” score plots, Hubbard (H) individuals are represented in turquoise and Ross (R) individuals in purple. The sex of the chicks: male (M, blue) and female (F, red) are represented in the “b” and “e” score plots. The individuals from the delayed group (D, orange triangle) and control group (C, light blue circle) are represented in the “c” and “f” plots.

a. Hubbard Classic

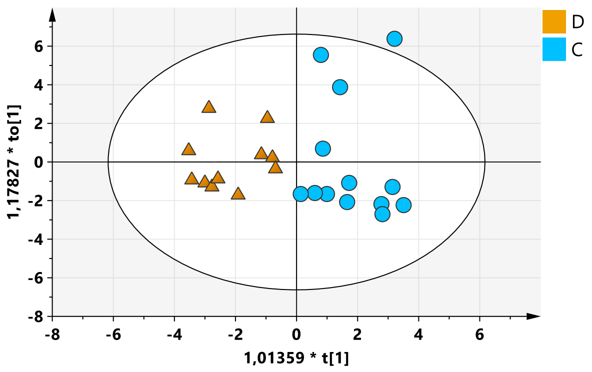

Metabolites	VIP	Contribution
Caffeate	1.35	1.62
Arabitol	1.32	1.54
2-Deoxyglucose	1.29	-1.72
Trigonelline	1.27	1.51
Acetyllysine	1.24	1.63
Cystathionine	1.23	1.43
Taurine	1.18	-1.45
Erythriol	1.18	1.25
Citramalate	1.03	1.01
2-Hydroxybutyrate	1.01	-0.94
Asparagine	0.97	0.16
Arginine	0.95	0.48
Glutamine	0.90	0.04
Serine	0.86	0.05
4-Guanidinobutanoate	0.85	0.51
Phosphoserine	0.81	0.62
Norepinephrine	0.70	0.61
Myristate	0.67	0.60
Palmitate	0.57	0.12
Cadaverine	0.38	0.22
Glucoseamine 6-phosphate	0.23	-0.09

R^2Y_{cum} = 0.78 ; Q^2_{cum} = 0.68 ; 1p + 1o ;
CV-ANOVA: 1.71×10^{-4}

b. Ross 308

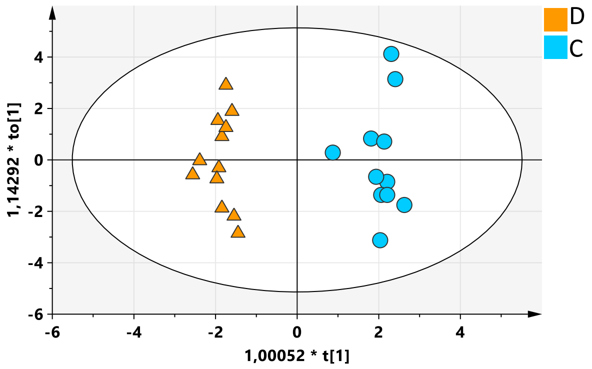

Metabolites	VIP	Contribution
Xanthosine	1.44	1.94
Uracile	1.44	-1.88
Inosine	1.42	1.85
Guanosine	1.27	1.53
Adenosine	1.25	1.47
Gluconate	1.22	1.36
Ferulate	1.08	1.09
Acetylglutamate	1.07	-0.98
4-Coumarate	1.05	-1.08
1-Oleyl-rac-glycerol	0.92	0.81
Acetylphenylalanine	0.88	-0.70
Malate	0.81	-0.65
2-hydroxy 4-methylthiobutyrate	0.76	0.50
Nicotinamide	0.50	0.22
Pantathenate	0.49	0.22
Leukotriene B4	0.42	0.16
Indoxylsulfate	0.40	0.15
Azelate	0.32	0.09

R^2Y_{cum} = 0.97 ; Q^2_{cum} = 0.93 ; 1p + 2o ;
CV-ANOVA: 2.20×10^{-8}

Fig. S2 OPLS-DA models adjusted to Hubbard (a) and Ross (b) chick fecal metabolomes at 12 days of age analyzed by **LC-HRMS**. The individuals from the delayed group (D, orange triangle) and control group (C, light blue circle) are represented in the score plots at the top of the figure. The fecal metabolites included in the Hubbard and Ross OPLS-DA models are tabulated with their variable importance in projection (VIP) and their contribution in the models. A negative contribution score indicates a contribution of the variable to the delayed group while a positive score is indicative of a contribution to the control group. The performance characteristics of each OPLS-DA model are under the metabolites tables (p = predictive component, o = orthogonal component).

a. Hubbard Classic

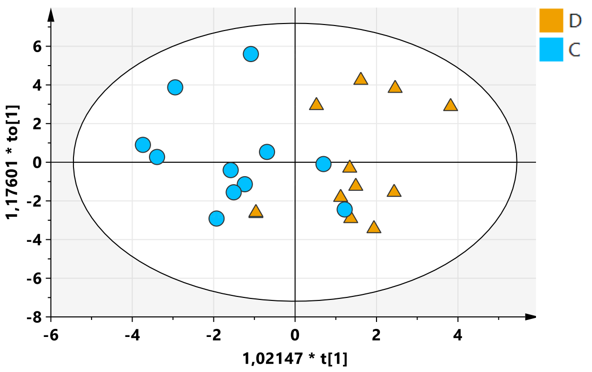

b. Ross 308

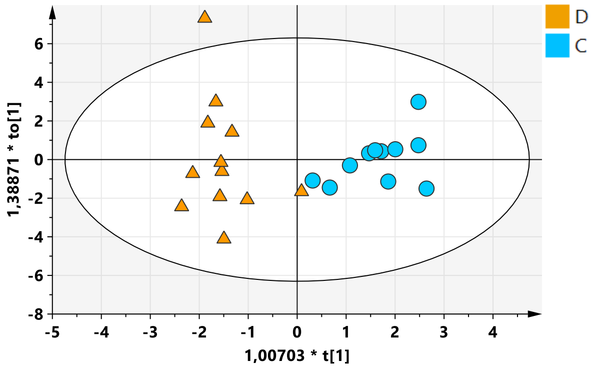

Metabolites	VIP	Contribution
2-Hydroxy-3-(4-hydroxy-3-methoxyphenyl) propanoate	1.33	1.34
Hydroxymandelate	1.31	1.35
Vanillylmandelate	1.25	1.25
Aconitate	1.24	1.18
Azelate	1.15	1.04
2-Methylisocitrate	1.14	1.12
3-Hydroxybutyrate	1.05	-1.09
Diethyl methylmalonate	1.04	1.07
Methylmalonate	1.01	0.87
2-Hydroxy 3-methylbutanoate	0.94	-0.37
2-Hydroxy 4-methylpentanoate	0.87	-0.31
Succinate	0.86	-0.13
Guaiacol	0.80	0.05
3-Phenyllactate	0.79	-0.04
Dodecanoate	0.62	0.41
Decanoate	0.55	0.10
Palmitate	0.49	-0.09

R^2Y_{cum} = 0.62 ; Q^2_{cum} = 0.48 ; 1p + 1o ;
CV-ANOVA: 0.016

Metabolites	VIP	Contribution
Pyruvate	1.39	-1.45
3-Hydroxyisobutyrate	1.39	-1.72
Lactate	1.37	-1.39
Tetracosanoate	1.28	-1.10
3-Hydroxypropanoate	1.27	-1.23
Succinate	1.25	-1.16
Dodecanoate	1.24	-1.14
Thymine	1.20	-1.08
Maleate	1.06	0.77
3-Hydroxy 2-methylbutanoate	0.95	-0.81
Vanillate	0.62	0.22
Hexanoate	0.59	-0.25
Ketoleucine	0.55	-0.29
Gentisate	0.47	-0.12
Phosphate	0.31	-0.07
Mandelate	0.26	-0.09
Sebacic acid	0.22	-0.12

R^2Y_{cum} = 0.86 ; Q^2_{cum} = 0.74 ; 1p + 2o ;
CV-ANOVA: 5.00×10^{-4}

Fig. S3 OPLS-DA models adjusted on Hubbard (a) and Ross (b) chick fecal metabolomes at 12 days of age analyzed by **GC-MS**. The individuals from the delayed group (D, orange triangle) and control group (C, light blue circle) are represented in the score plots at the top of the figure. The fecal metabolites included in the Hubbard and Ross OPLS-DA models are tabulated with their variable importance in projection (VIP) and their contribution in the models. A negative contribution score indicates a contribution of the variable to the delayed group while a positive score is indicative of a contribution to the control group. The performance characteristics of each OPLS-DA model are under the metabolites tables (p = predictive component, o = orthogonal component).
